# Transcriptomic Study Reveals Recovery of Impaired Astrocytes Contribute to Neuroprotective Effects of Danhong Injection Against Cerebral Ischemia/Reperfusion-Induced Injury

**DOI:** 10.3389/fphar.2018.00250

**Published:** 2018-03-27

**Authors:** Jing Qian, Xiaoping Zhao, Weiting Wang, Shujing Zhang, Zhuping Hong, Xiaoling Chen, Zhuanyou Zhao, Chunhua Hao, Chenchen Wang, Shihai Lu, Buchang Zhao, Yi Wang

**Affiliations:** ^1^Pharmaceutical Informatics Institute, College of Pharmaceutical Sciences, Zhejiang University, Hangzhou, China; ^2^College of Preclinical Medicine, Zhejiang Chinese Medical University, Hangzhou, China; ^3^State Key Laboratory of Pharmacokinetics and Pharmacodynamics, Tianjin Institute of Pharmaceutical Research, Tianjin, China; ^4^Shandong Danhong Pharmaceutical Co., Ltd., Heze, China

**Keywords:** cerebral ischemic/reperfusion injury, Danhong injection, microarray, astrocyte, inflammation, brain–blood barrier

## Abstract

Danhong Injection (DHI) is widely used in clinics for treating cardiovascular and cerebrovascular diseases in China. However, the mode of action of DHI for neuroprotection remains unclear. In the present study, we deemed to investigate the effects of DHI on a rat model of cerebral ischemia/reperfusion injury (IRI) with an emphasis on its regulated gene profile obtained from microarray assays. Firstly, we showed that a 14-day DHI treatment effectively ameliorated severity of neurological deficits, reduced size of ischemic damage, improved status of oxidation stress, as well as systemic inflammation for IRI rats, along with which was a pronounced reduced cell infiltration in the area of periaqueductal gray matter. Secondly, bioinformatic analyses for the 429 differentially expressed genes (DEGs) regulated by DHI treatment pointed out ECM–receptor interaction, neuroactive ligand–receptor interaction, and endocytosis as the top three biological processes, while Toll-like recptor 4 (TLR4) as the most relavant singaling molecule. Lastly, we provided evidences showing that DHI might directly protect primary astrocytes from oxygen and glucose deprivation/re-oxygenation (OGD/Re) injury, the effects of which was associated with LAMC2 and ADRB3, two DEGs related to the top three biological processes according to transcriptomic analysis. In conlusion, we reported that DHI might work through maintaining the integrity for brain–blood barrier and to regulate TLR4-related signaling pathway to diminish the inflammation, therefore, effectively improved the outcomes of IRI. Our findings suggested that the attenuated astrocytic dysfunction could be a novel mechanism contributing to the neuroprotective effects of DHI against cerebral ischemia/reperfusion-induced damage.

## Introduction

Ischemic stroke or cerebral ischemia has become the leading cause of mortality and morbidity worldwide. As the currently first line of therapy, the restoration of blood supply/reperfusion to the occluded cerebral area may cause neurovascular reperfusion injury, leading to an even worse disease outcome (Jung et al., 2010; Kalogeris et al., 2016). Besides, although drugs that targeting for inflammation, oxidative stress, apoptosis, and other pathophysiological mechanisms have been developed, there still lacks of effective neuroprotective agents for the prevention and treatment of cerebral ischemia (Benjamin et al., 2017).

Danhong injection, chiefly composed of two herbs *Salviae miltiorrhiza Bunge* (Danshen in Chinese) and *Carthamus tinctorius L.* (Honghua in Chinese), is used extensively in China for the prevention and treatment of various vascular diseases including occlusive vasculitis, coronary diseases, and cerebral infarction, etc. (He et al., 2012; Chen Y. et al., 2014; Guo et al., 2014). There are supportive experimental evidences showing the efficiency of DHI in the treatment of cerebral ischemic disorder. *In vivo*, the pretreatment with DHI could ameliorate neurological score, cerebral infarction, and brain edema in rats with cerebral IRI (Guo et al., 2014; Wang et al., 2014). *In vitro*, DHI could protect mouse Neuro-2A cells from H_2_O_2_ wounding, along with activation of Nrf2/ARE signaling pathway (Guo et al., 2014). Recent pharmacological investigations showed that DHI could inhibit hepatic fatty acid synthesis (Chen J. et al., 2014) and inhibit inflammatory NF-kappaB activation (Jiang et al., 2015). It is also reported that different active compounds of DHI may exert synergistic cardioprotective effects through anti-inflammation, anti-oxidation, and anti-apoptosis (Guan et al., 2013). We have recently showed an anti-thrombotic effect of DHI and its major constituents on a zebrafish model of arachidonic acid (AA)-induced thrombosis (Qi et al., 2017). However, the exact mechanism of action for neuroprotective effects of DHI is poorly understood.

In fact, the unclear therapeutic target is a common problem faced by the majority of herbal medicines (Layne and Ferro, 2017). Although extensively clinical applications have confirmed the efficacy of herbal medicine, there still lacks of scientific explorations about their pharmacological activities. As a valuable method to investigate holistic view of gene expression profiles in biological system, transcriptomic techniques have been widely applied in pathophysiological research of disease and clinical diagnosis. However, there is few reports to dissect mechanism of action of herbal medicine by transcriptomic analysis.

The objective of the current study was to investigate the mode of action of DHI for the treatment of cerebral IRI by transcriptomic approach. Neurological deficits grading, cerebral infarct size, oxidative stress status, and inflammatory cytokines production were measured to evaluate the therapeutic effect of DHI. Microarray-based transcriptomic analysis and network pharmacology approaches were used to study DEGs and their related biological functions after DHI treatment. The findings might provide some molecular insights into the mechanism of action of DHI.

## Materials and Methods

### MCAO-Induced Focal Cerebral Ischemia and Reperfusion (I/R) Injury in Rats

Specific pathogen-free (SPF) male Sprague-Dawley (SD) rats, weighing 260–300 g, were obtained from Charles River Laboratories (Beijing, China. Laboratory animal certificate: SCXK 2012-0001). The animal care and use protocols were approved by Tianjin Institute of Pharmaceutical Research. Focal cerebral IRI was produced by 2 h-MCAO. In brief, after anesthetized with isoflurane, the right common carotid artery (CCA) of the rat was isolated and the branches of external carotid artery (ECA), i.e., occipital artery, superior thyroid artery, lingual artery, and maxillary artery were ligated and cut. Next, 3-0 monofilament nylon suture was inserted via the proximal ECA into the internal carotid artery and thence into the circle of Willis. The length of the nylon suture was about 18–20 mm from the bifurcation of CCA/ECA. Two hours after the occlusion, the nylon suture was removed to restore blood supply to the MCA area by reperfusion. Ten minutes after the surgery, neurological deficits were evaluated and scored. For sham-operated rats, the ECA was isolated but no filament was introduced.

### Drug Administration

Danhong injection was provided by Buchang Pharmaceutical Co. Ltd. (Heze, Shandong, China). Different dosages of DHI, calculated according to the clinical consumption as 0.4, 0.8, and 1.6 mL/kg, were chosen and prepared in normal saline (NS). Animals were randomly divided into five groups: (1) sham-operated (Sham) group; (2) I/R group; (3) 0.4 mL/kg DHI treatment group; (4) 0.8 mL/kg DHI treatment group; and (5) 1.6 mL/kg DHI treatment group. Rats were tail vein administered with 500 μL DHI/NS containing indicated amount of DHI daily, starting after the surgery till 14 days when animals were sacrificed for further studies. In the case of the sham-operated group, equal volume solvent was administered in the same manner.

### Neurological Deficits Grading

Neurological deficits of rats were assessed 14 days after MACO in a double-blind manner by two independent examiners. The range of the score was between 0 and 16 according to a modified neurological severity scoring criteria (Chen et al., 2001), for which a higher score represented a worse neurological condition. The rats with neurological deficits scoring above 8 points were considered as successful modeling for I/R injury. All the sham-operated rats had the score of 0.

### Cerebral Infarction Size

The whole brain was removed and continuously cut into 2-mm thickness cerebral sections along the coronal plane. After staining with 2% 2,3,5-triphenylte-trazoliumchloride (TTC) solution in the dark for 5 min at 37°C, the images for the sections were recorded with a digital camera (COOLPIX955, Nikon, Japan). The infarction size was analyzed with Image (version 4.0) and expressed as a percentage of fraction area over the global brain.

### Histopathological Changes

The whole brain was fixed with 4% paraformaldehyde before tissues within and surround the infarcted area were coronal incised and embedded with paraffin. Five-micrometer sections were sliced for hematoxylin and eosin (HE) staining and microscopy (TS100, Nikon, Japan).

### Measurement of Oxidative Stress and Cytokine Release in Serum

Blood from abdominal aorta was collected and the serum sample was prepared thereof. The content of malondialdehyde (MDA); activities of superoxide dismutase (SOD), glutathione peroxidase (GPx), and Monoamine oxidase (MAO); and levels of reduced glutathione (GSH) were measured by using commercial available kits (Nanjing Jiancheng Bioengineering Institute, Nanjing, China).

The concentrations of cytokines in the serum samples were measured using enzyme-linked immunosorbent assay (ELISA) kits for IL-1β, IL-6, and TNF-α according to the manufacturer’s instructions (Bio-Swamp, Wuhan, China).

### Microarray Processing and Data Analysis

We profiled the gene expression in the infarcted hemispheres of Sham, I/R, and DHI-treated (0.8 g/kg) groups (*n* = 3) using Agilent SurePrint G3 Rat GE Microarray supported by Oebiotech^1^ (Shanghai, China). Total RNA was isolated from rat brain tissue using mirVanaTM RNA Isolation Kit and purified using the RNeasy Mini kit (Qiagen, Chatsworth, CA, United States) and RNase-free DNase Set (Qiagen, Chatsworth, CA, United States) following the manufacturer’s instructions. The sample labeling, microarray hybridization, and washing were performed based on the manufacturer’s standard protocols. The arrays were eventually scanned (Agilent Scanner G2505C, Agilent Technologies Co. Ltd., Palo Alto, CA, United States) with settings for one-color scans. The raw microarray data are available through the National Center for Biotechnology Information’s Gene Expression Omnibus (GEO series accession number: GSE106680^2^).

Genes measured by the probe sets with a fold change (FC) greater than a pre-defined threshold and a *p*-value less than a pre-defined threshold were considered as differentially expressed. In this study, genes with FC > 1.5 and *p* < 0.05 were defined as DEGs. Venny 2.1.0^3^ was used to construct Venn diagram. MEV 4.6.0 software was applied for hierarchical clustering analysis combined with heatmap construction to estimate the overall reproducibility and variation of three replicates within each group and the differences among the sham, I/R, and DHI groups. Functional enrichment analysis was carried out by using DAVID software tool^4^, with that pathway enrichment and functional processing categories were generated based on KEGG database and annotations of Gene Ontology, respectively. In addition, GeneMANIA^5^ was employed to generate protein-interaction network (Warde-Farley et al., 2010).

### Quantitative RT-PCR (qRT-PCR)

The expressions of four representative genes of CD44, LAMC2, ADRB3, and LDLR were validated by qRT-PCR. Total RNA from brain tissue was prepared as described for microarray assay, whereas primary astrocytes were extracted with the RNA simple Total RNA Kit (CWBIO, Beijing, China). First-strand cDNA was synthesized from 1 μg total RNA (DNase-treated) using the HiFiScript cDNA synthesis kit (CWBIO, Beijing, China). RT-PCR was performed with an authorized Thermal Cycler (Eppendorf, Hamburg, Germany). qPCRs were then performed with 1 μL cDNA using UltraSYBR Mixture (CWBIO, Beijing, China) with the CFX96 Touch Real-Time PCR Detection System (CFX96, Bio-Rad, Hercules, CA, United States). Primers were provided by Sangon Biotech (Shanghai, China) and were described in **Table 1**. β-Actin was amplified as an endogenous reference gene. The relative expression level was calculated by ΔΔCt method with Bio-Rad CFX Manager (Bio-Rad, Hercules, CA, United States) and expressed as fold of change to control.

**Table 1:** Primer sequences for qPCR.

Targets	Forward primer (5′–3′)	Reverse primer (5′–3′)
β-Actin	CCCTGGCTCCTAGCACCAT	GAGCCACCAATCCACACAGA
CD44	CCTCGCATCCAACACCTCCCAC	TGTAGCGGGTGCCATCACGG
LAMC2	ACATTGAGTGTGCCGACTGC	CCTCTGTCTCAGGCATCACG
ADRB3	CGTCTTCTGTGCAGCTACGG	GGACGCTCACCTTCATAGCC
LDLR	AGATCTACAGCGCCGTGATG	CATGGATCCAGTCTACCGCC


### Effect of DHI on Primary Astrocytes in a Model of Oxygen and Glucose Deprivation/Reoxygenation (OGD/Re)

Primary astrocytes were isolated according to previous published protocols (Kajitani et al., 2012; Morioka et al., 2014). In brief, the whole brain of 1-day-old neonatal SD rat was removed for cerebral cortices dissection. Afterward, the tissue was applied for mechanically dissociation, trypsin and DNase treatment, and 40 μm cell strainer filtration. In the end, the cells were collected and resuspended in DMEM containing 10% FBS and 1% penicillin–streptomycin (complete culture medium). Unless specified, all of the cell culture reagents were purchased from Invitrogen (Shanghai, China). Cells were cultured in a 75 cm cell culture flask (Corning, NY, United States) at a density of 3 × 10^5^ cells/mL at 37°C in a 5% CO2/95% air atmosphere for 10–14 days. Finally, the cell flacks were shaken at 180 rpm (37°C, 18 h) and remaining cells were considered as astrocytes. The purity of astrocytes was around 90% as exanimated by the fluorescent staining of the cell marker GFAP.

Oxygen and glucose deprivation/reoxygenation (OGD/Re) injury was induced for the astrocytes (Zhu et al., 2017). In brief, 2 × 10^5^ cells/mL in serum-free Earle’s solution were seeded in wells of either 6- or 96-well plate and were allowed to grow for 24 h before the cell culture flasks were placed in a sealed chamber loaded with mixed gas containing 95% N_2_ and 5% CO_2_ at 37°C. After 6 h of OGD, the cell culture medium was replaced with DMEM containing 10% FBS and 1% penicillin–streptomycin and the cells were continued cultured for 24 h at 37°C with 5% CO_2_ to build up reperfusion. For the DHI treatment, DHI with indicated dosage was added into the culture medium to throughout the process of OGD/R injury.

In the end of experiment, cells were either applied for MTT assay or collected for RNA extraction and RT-qPCR. For cell viability assay of astrocytes, cells were cultured in 96-well plates for 24 h at a density of (2 × 10^4^) cells/well, with or without the supplement of DHI at the dosage of 1.25, 2.5, 5, and 10 μL/mL, respectively. After OGD/Re injury, the media were removed and 5 mg/mL MTT reagents (M2128, Sigma-Aldrich, Santa Anta, CA, United States) in PBS were loaded to all respective wells followed by 4 h incubation in a CO_2_ incubator at 37°C. Further, MTT solution was removed and 100 μL of DMSO (Darmstadt, Germany) was used to dissolve formazan crystals. Finally, the microtiter plates were agitated for 10 min on an orbital plate shaker at 37°C before the absorbance at 570 nm of each well was measured by Microplate Reader (Infinite M1000, Tecan, Switzerland).

### Statistical Analysis

All values are expressed as the means ± SD. One-way ANOVA was used to analyze differences among groups. Statistical analysis was performed using GraphPad Prism. *p*-Values of <0.05 were considered statistically significant.

## Results

### DHI Ameliorates Cerebral Infarction and Neurological Deficits in I/R Rats

**FIGURE 1:**
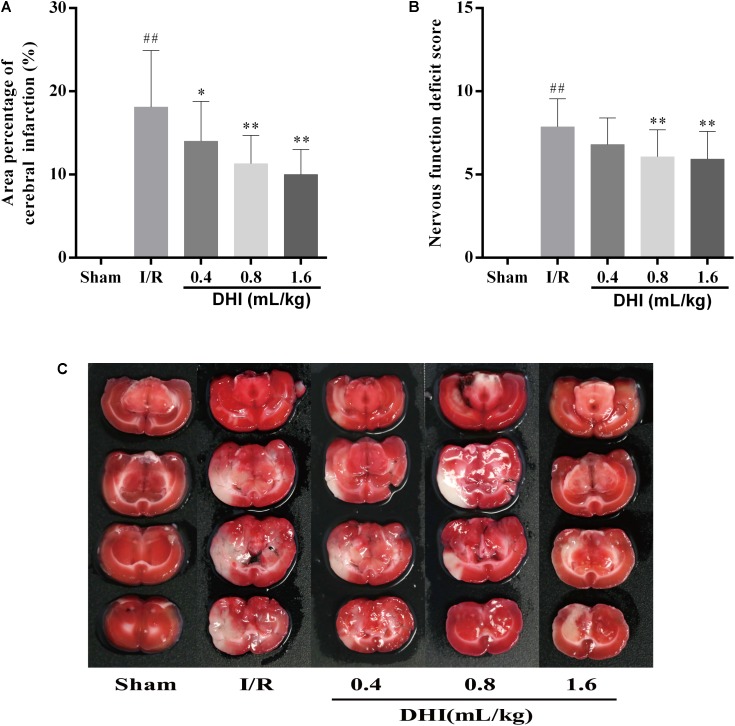
DHI reduced the area of brain damage and improved neurological functional outcome following cerebral IRI. **(A)** Mean area percentage. **(B)** Neurological deficits grading score. **(C)** Representative TTC stained serial coronal brain sections. Values were expressed as mean ± SD. ^##^*p* < 0.01 compared with the Sham group; ^∗^*p* < 0.05, ^∗∗^*p* < 0.01 compared with the IRI group.

Area percentage of cerebral infraction and the neurological function deficit score were measured. As shown in **Figures 1A,C**, while rats in Sham group showed no evidence for neuron injury, I/R group appearing 18.1 ± 6.8% area of cerebral infraction. The treatment of DHI reduced the infraction area in a dose-depended manner, i.e., the 0.4-, 0.8-, and 1.6 mL/kg DHI treatment group appearing 14.0 ± 4.8% (*p* < 0.05), 11.3 ± 3.4% (*p* < 0.01), and 10.0 ± 3.0% (*p* < 0.01), respectively. The scoring of neurological function showed similar effects. The application of DHI at the dosage of 0.4-, 0.8-, and 1.6 mL/kg reduced the neurological deficit score of the I/R from 7.87 ± 1.68 to 6.8 ± 1.61, 6.07 ± 1.62 (*p* < 0.01), and 5.93 ± 1.67 (*p* < 0.01), respectively (**Figure 1B**).

### DHI Presents Anti-oxidative Property in I/R Rats

**FIGURE 2:**
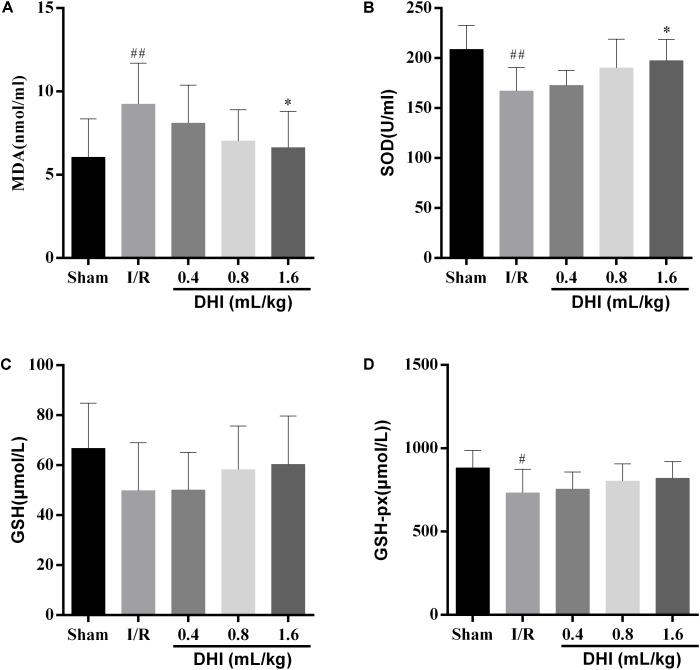
DHI prevent the IRI rats from releasing oxidative stress-related enzymes. **(A)** MDA, **(B)** SOD, **(C)** GSH, and **(D)** GSH-PX activities were determined by colorimetric method. Values were expressed as mean ± SD. ^#^*p* < 0.05, ^##^*p* < 0.01 compared with the Sham group; ^∗^*p* < 0.05 compared with the IRI group.

The activities of endogenous antioxidant enzymes of MDA, SOD, GSH, and GSH-PX were measured. As shown in **Figure 2**, comparing with the sham-operated rats, the I/R ones showed significantly higher levels of the MDA peroxidation (9.24 ± 2.47 versus 6.06 ± 2.3, *p* < 0.01) but significant lower levels of SOD (167.1 ± 23.4 versus 208.9 ± 24.0, *p* < 0.01). At the time of examination, there were no differences for the releasing of GSH and GSH-px (733.0 ± 141.2 versus 883.7 ± 102.8, *p* < 0.05) between the two groups. The treatment of DHI resulted in alternated releasing of MDA and SOD to the levels similar to sham-operated group in a dose-dependent manner. When at the dosages of 0.8- and 1.6 mL/kg, the effects were of statistically significant (*p* < 0.01).

### DHI Suppresses Inflammatory Cytokines Releasing Caused by Cerebral I/R Injury

**FIGURE 3:**
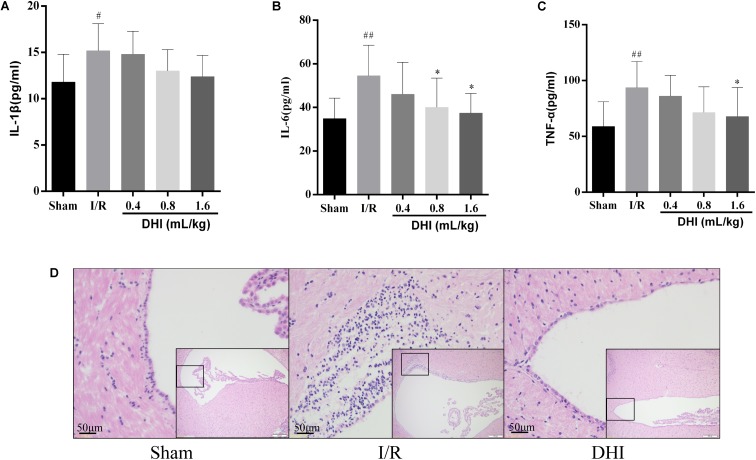
DHI suppresses inflammation related to cerebral IRI. **(A–C)** Serum levels of IL-1β, IL-6, and TNF-α, respectively. **(D)** Representative H&E staining section of brain tissues. Values were expressed as mean ± SD. ^#^*p* < 0.05, ^##^*p* < 0.01 compared with Sham group; ^∗^*p* < 0.05 compared with IRI group.

Next, we evaluated the effect of DH on inflammation resulted from the MACO surgery. As illustrated in **Figure 3**, the inflammatory mediators of blood sample including IL-1β, IL-6, and TNF-α in model group increased significantly compared with that in sham group (*p* < 0.05 or *p* < 0.01). Treatment with DHI 0.8 and 1.6 mL/kg for 14 days inhibited the releasing of these inflammatory cytokines. IL-1β, IL-6, and TNF-α levels declined from 15.2 ± 2.9, 54.6 ± 14.0, and 93.7 ± 23.4 in model group to 13.0 ± 2.3, 40.1 ± 13.4 (*p* < 0.05), and 71.4 ± 23.0 in DHI 0.8 mL/kg group and 12.4 ± 2.3 (*p* < 0.05), 37.4 ± 9.0 (*p* < 0.05), and 67.8 ± 26.0 (*p* < 0.05) in DHI 1.6 mL/kg group, respectively (**Figures 3A–C**). Notably, in I/R injury rat, HE staining for the brain tissue showed infiltrations of inflammatory cells in the area of periaqueductal gray matter (**Figure 3D**). The application of DHI almost diminished this phenomenon, HE staining results for the same area showed rare inflammatory cell infiltration, which was similar to sham-operated rats.

### Microarray Data Analyses

**FIGURE 4:**
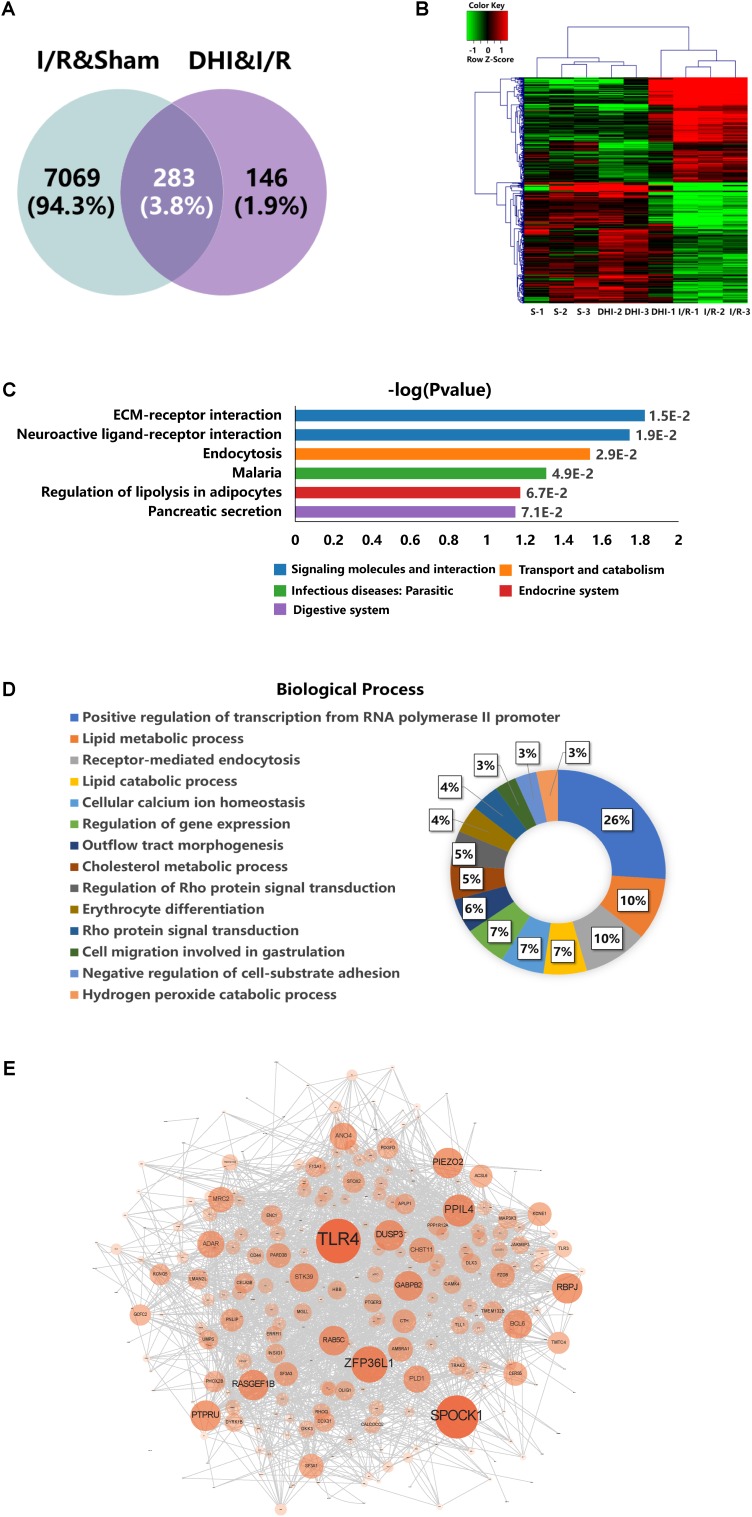
DEGs profiling analyses. **(A)** Venn diagram summary of the DEGs between Sham versus IRI groups and DHI versus IRI groups. **(B)** Hierarchical clustering analysis and heatmap of the correlation coefficients between altered gene expression profiles. S1–3 represent the three biological replicates for Sham group, DHI1–3 for DHI group, and I/R1–3 for IRI group, respectively. **(C)** Top KEGG pathways enriched with DEGs and their corresponding *p*-values. **(D)** Doughnut chart presentation for the distribution of altered genes in biological processes. The individual biological process was distinguished with color and percentage value. **(E)** Protein–protein interaction network generated from GeneMANIA analysis. The size of each node presents the degree of connectivity in PPI network. Large node shares more connection to other nodes.

By applying the cutoffs of *t*-test *p-*value < 0.05 and FC > 1.5, the DEGs were selected. As shown in **Figure 4A**, 7352 DEGs were identified in I/R versus Sham groups (I/R and Sham), whereas 429 DEGs were identified in DHI versus I/R groups (DHI and I/R). Venn diagram showed that 283 out of the 429 DEGs responding to DHI treatment were related to DEGs caused by I/R injury. Hierarchical clustering analysis showed that the expression profiles of the 429 DEGs in Sham and DHI groups were dramatically different to that of the I/R group (**Figure 4B**).

The KEGG pathway and biological processes for the identified 429 DEGs involved were depicted. As presented in **Figure 3C**, the top three pathways enriched from KEGG database were ECM–receptor interaction, neuroactive ligand–receptor interaction, and endocytosis. The DEGs contributed to these three pathways were listed in **Table 1**. As presented in **Figure 4D**, the result of biological process showed that 26% of the DEGs were involved in positive regulation of transcription, 22% were involved in regulating lipid metabolic process among which 10% for lipid metabolic process, 7% for lipid catabolic process, and 5% for cholesterol metabolic procss, 10% were involved in regulating endocytosis and others involving regulation of Rho protein signal transduction, cell migration and adhesion, cellular calcium ion homeostasis, outflow tract morphogenesis, and so on.

Protein-interaction network analysis, as detcted by the size and depth of each node which is positively correlated to the degree of protein connection to others, identified TLR4 as the major relavant signaling molecule (**Figure 4E**). The others include SPOCK1, ZFP36L1, PPIL4, etc. (**Figure 4E**).

### Validation of Gene Expression

**FIGURE 5:**
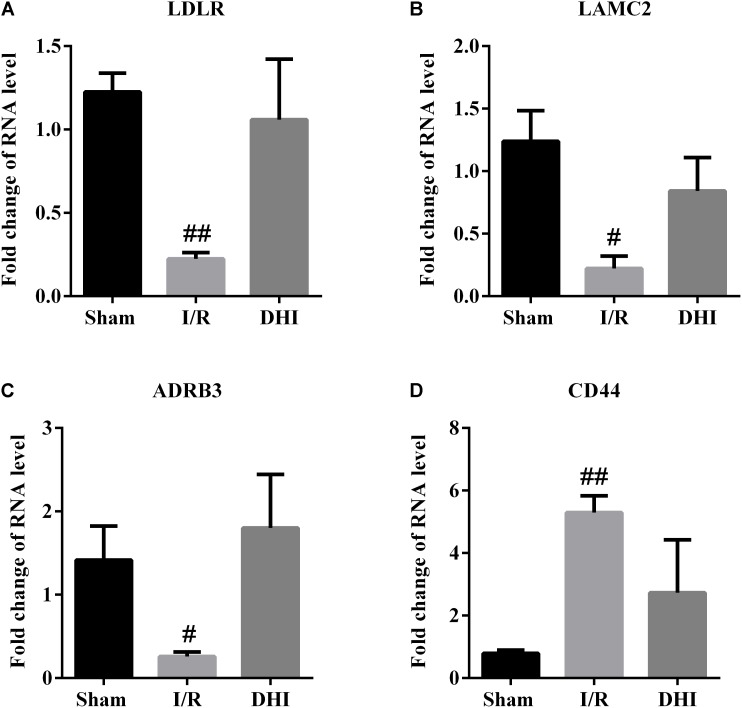
RT-qPCR validation for microarray data. The expressions of **(A)** LDLR, **(B)** LAMC2, **(C)** ADRB3, and **(D)** CD44 in rat brains were examined by RT-qPCR. Data were expressed as fold of changes compared to Sham group. ^#^*p* < 0.05, ^##^*p* < 0.01 compared with Sham group.

To validate the microarray results, the expression of CD44, LAMC2, ADRB3, and LDLR as DEGs representative for the top three pathways predicted through DAVID was validated by qRT-PCR. The results from qRT-PCR assay were in accordance well with the array data, that the I/R injury resulted in down-regulated expressions of LAMC2, ADRB3, and LDLR, along with up-regulated expression of CD44 and that the DHI treatment adjusted their expression to Sham group level (**Figure 5**).

### DHI Protects Astrocytes From OGD/Re-Induced Injury Associated With LAMC2 and ADRB3

**FIGURE 6:**
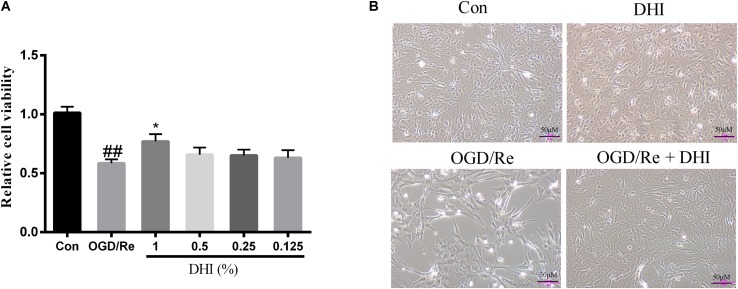
DHI protects astrocytes from OGD/Re-induced cell death. **(A)** MTT assay. Data were presented as relative cell viability compared to control (mean ± SD from three independent experiments). ^##^*p* < 0.01 compared with control group; ^∗^*p* < 0.05 compared with OGD/Re group. **(B)** Representative light microscope images of astrocytes exposed to OGD/Re injury with or without 1% DHI treatment.

An oxygen and glucose deprivation/reoxygenation model in primary cultured astrocytes was applied to mimic the IRI *in vitro* with or without adding the DHI at indicated dosage immediately at the onset of OGD. The cell viability was detected by both light-converted microscopy and MTT assay. Results showed that while OGD/Re led to astrocytes cell shrink and death, the treatment of DHI protected the astrocytes from cell death in a dose-dependent manner (**Figures 6A,B**).

**FIGURE 7:**
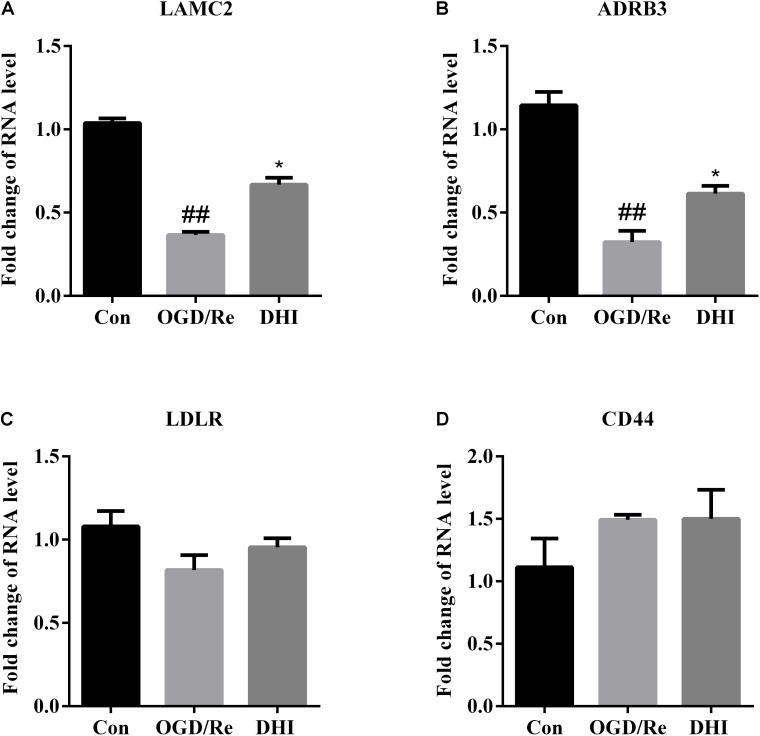
DHI regulates Lamc2 and Adrb3 gene expression in OGD/Re-injured astrocytes. The expressions of **(A)** LAMC2, **(B)** ADRB3, **(C)** LDLR, and **(D)** CD44 were determined by qRT-PCR. Data were expressed as fold of changes compared to control cells. ^##^*p* < 0.01 compared with control; ^∗^*p* < 0.05 compared with OGD/Re group.

We also checked expression of CD44, LAMC2, ADRB3, and LDLR, the DEGs that were validated as regulatory targets for DHI in IRI rats by both microarray and qRT-PCR assays. Results showed that the expression patterns of LAMC2 and ADRB3 in astrocytes were similar to those in rats. As indicated in **Figure 7**, when the OGD/Re injury resulted in down-regulated expressions of LAMC2 and ADRB3 in astrocytes, the treatment of DHI recovered their expressions comparable to control levels.

## Discussion

In this study, we reported that a 14-day application of DHI effectively improved the outcomes of cerebral IRI, as evidenced by the neurological deficits grading, cerebral infarction size, status for oxidative stress, and inflammation in a rat model of MACO plus reperfusion injury. In line with microarray data, we showed that DHI protect astrocytes from ODG/Re. Our study provided a genetic platform applicable for investigating the mode of action of DHI; it also gave clues to recognize therapeutic targets and explore novel strategies as potential treatments to IRI.

A large number of pathologic processes underlie reperfusion injury, which mainly include the oxidative stress, calcium overload, as well as inflammation (Kalogeris et al., 2012; Terasaki et al., 2014). The infiltration of inflammatory cells, such as neutrophils, macrophages, lymphocytes, etc. may last for a long period of time (Hu et al., 2012). As a consequence, patients may suffer from neuron functional deficiency, as well as high risks for local or systemic inflammation (Westendorp et al., 2011; Becker and Buckwalter, 2016; Santos Samary et al., 2016). Furthermore, it was recently identified that the gene expression patterns vary over the course of acute IRI (Wang et al., 2017). In our model rats, there existed pronounced neuron functional deficiency, oxidative stress, and inflammation at 14 days after I/R injury (**Figures 1**–**3**). Notably, the cell infiltration was most relevant in the area of periaqueductal gray matter (**Figure 3D**), which highlighted the existing of the breaking down of brain–blood barrier (BBB).

The therapeutic effect for DHI in treating cerebral I/R injury have been evaluated in several studies, including evidence-based clinical studies as well as both *in vivo* and *in vitro* experimental settings (He et al., 2012; Guo et al., 2014; Wang et al., 2014). DHI have shown pronounced protected effects of in the acute phase of the disease (from hours up to 7 days). Take for example, by applying a rat model of temporary MACO, Wang et al. (2014) showed pre- as well as post-treatment of DHI had the neuroprotective efficacy at both 24 and 72 h after surgery, associated with which were the protection on the BBB and the reversal of neutrophil infiltration. In addition, Guo et al. (2014) showed that the mode of neuroprotective action of DHI might be related to the activating of Nrf2/ARE signaling pathway. In the present study, we focused on checking the effects of DHI in the process of the cerebral I/R injury. We provided solid data showing the prolonged application of DHI to 14 days improved the outcome of rats from cerebral I/R injury in the recovery process (**Figures 1**–**3**). Importantly, we performed microarray assay and provided global transcriptional data, which enabled us to explore molecular mechanisms contributing to protective effects of DHI against cerebral I/R injury. Although bioinformatics approaches, including proteomic analysis (Cui et al., 2017) and systematic survey for transcription factors (TFs) changes (Wei et al., 2016) have been applied, ours is to focus on the transcriptomic analysis, and on a late time point of 14 days.

In the present study, we prevailed ECM–receptor interaction, neuroactive ligand–receptor interaction, and endocytosis as the top three biological processes related to DHI treatment caused by I/R injury (**Figure 4C**). The main DEGs include CD44, LAMC2, LDLR, ARBD3, etc. (**Table 2**). CD44 is a cell adhesion molecule known to be involved in endothelial cell recognition, lymphocyte trafficking, and regulation of cytokine gene expression in inflammatory diseases (Xu et al., 2015). In ischemic lesions, the expression of CD44 could be detected in microglia/macrophages and brain capillaries (Al Qteishat et al., 2006). Blockade of CD44 could ameliorate ischemic brain injury related to the reduction of soluble IL-1β expression (Wang et al., 2001). The gene LAMC2 encodes gamma2 chain of laminin 5, an epithelial cell-specific extracellular matrix protein, which might be related to the anchoring of astrocytes to the microvascular wall (Wagner et al., 1997). Interestingly, recent study showed that Lmc2 protein could interact with CD44 on cancer cell membrane (Sato et al., 2015), which indicated that during the process of IRI, laminin 5 might function as a ligand of CD44. The alternated level of low-density lipoprotein receptor (LDLR) was apparent in I/R brain (Rousselet et al., 2011). It might be involved in lipid raft associated neuron function and inflammatory response in IRI (Paciullo et al., 2017). Adrb3 encodes β3-AR, mainly expressed in white adipose tissue, but also in the heart, brain, lungs, and liver (Gao et al., 2017). Those genes and function therefore are novel targets of interests for further investigation.

**Table 2:** Top pathways prediction of DEGs by DAVID.

Pathway	*p*-value	DEGs
ECM–receptor interaction	0.015	CD44, LAMC2, LAMC3, VTN, THBS4, HMMR
Neuroactive ligand–receptor interaction	0.018	ADRB3, PTGER3, ADORA3, P2RY4, GRIK4, PRSS3, CHRNA5, CHRNB3, ADORA1, NTSR2, TAAR2
Endocytosis	0.029	ADRB3, LDLR, EPN3, PLD1, AP2A1, RAB5C, ACAP1, PML, PARD6G, GRK1


Inflammation is a pronounced phenomenon in the I/R-injured brain (Kalogeris et al., 2016). In addition to the local inflammation, the systemic inflammation may also exist. In the present study, we showed three lines of evidence indicating the anti-inflammatory effects of DHI. (1) DHI inhibited the production of systemic inflammatory cytokines of IL-1β, IL-6, and TNF-α. (2) DHI attenuated the inflammatory cell infiltration in the brain especially in the area around periaqueductal gray matter, where the MACO and reperfusion took place. (3) Gene association network analysis based on DEGs identified from chip expression pointed out TLR4 as a key knot of responsive gene to DHI treatment after I/R injury. It has been shown that TLR4, via recognition of pathogen-associated molecular patterns (PAMPs) released from damaged neurons, attributes to the further brain damage (Marsh et al., 2009; Kapetanovic et al., 2015). In TLR4 knock out mice, the I/R injury is released (Caso et al., 2007). Therefore, it is likely that DHI may interfere with TLR4-related singling pathway, therefore inhibit the endogenous PAMPs triggered inflammation as a consequence of cerebral I/R injury. Since the inflammation may last for a long period of time and may affect the recovery of brain function, the regulatory effects of DHI on inflammation are essential for recovery.

The BBB, composed of endothelial, astrocytes, as well as microglial cells, accounts for the influx of inflammatory cells (Abbott et al., 2006) and affects the degree of brain damage and recovery (Dirnagl, 2012; Terasaki et al., 2014). The modulation of astrocyte functions as a part of maintaining BBB integrity has been reported applicable to treating ischemic stroke (Pan et al., 2016). In the current study, the anti-inflammatory effects of DHI could be partially explained by the enhancement of BBB function, as a result of which, the brain damage released or the repair enhanced. This assumption is supported by the DEGs analysis data that in the list of DEGs due to DHI treatment in IRI rats after 14 days treatment, ECM–receptor interaction, the neuroactive ligand–receptor interaction, and endocytosis were identified as most relevant biological processes.

Although the roles for listed DEGs, i.e., LAMC2, ARBD3, CD44, and LDLR, etc., are not fully illustrated for astrocytes in the scenario of cerebral I/R injury, our *in vitro* experiment showed solid data supporting the hypothesis that DHI have the protective effects on primary astrocytes in a ODG/Re model which mimic the effects for I/R injury. Our results are in line with the previous report that glial cells are the main player to drive preconditioning-induced BBB protection (Gesuete et al., 2011). Interestingly, while we check the expression patterns for the molecules identified from microarray data analysis, the expressions of LAMC2 and ADRB3 were down regulated due to ODG/Re injury. DHI significantly up-regulated their expression similar to control level.

Because so many different deleterious events participate in I/R, it is recognized that therapeutic approaches will be effective only when multiple pathologic processes are targeted. Our result put DHI as an ideal example for multiple targets (biological events)-interfering. In addition, by providing gene expression profile, we highlighted several novel targets that might be applicable to treat cerebral I/R injury in the recovery process, i.e., rebuild the BBB associate with astrocytes, regulating TLR4 signaling pathway, etc.

## Conclusion

Danhong injection effectively improved the outcomes of cerebral IRI in the recovery process, the molecular mechanisms of which may include maintaining the integrity for BBB and to regulate TLR4-related signaling pathway to diminish the inflammation and benefit for recovery.

## Author Contributions

ZZ, WW, and CH performed the animal studies. JQ and ZH performed the *in vitro* studies and participated in the figure preparation. YW and SZ performed the transcriptomic data analysis and participated in the figure preparation. XC, CW, and CH participated in the critical revision of the manuscript. YW, JQ, BZ, SL, and XZ led the project, oversaw the manuscript preparation, and wrote the manuscript.

## Conflict of Interest Statement

CW, SL, and BZ were employed by company Shandong Danhong Pharmaceutical Co., Ltd. The other authors declare that the research was conducted in the absence of any commercial or financial relationships that could be construed as a potential conflict of interest.

## Footnotes

1 http://www.oebiotech.com/

2 http://www.ncbi.nlm.nih.gov/geo/

3 http://bioinfogp.cnb.csic.es/tools/venny/

4 http://david.abcc.ncifcrf.gov

5 http://genemania.org/

## Abbreviations

DEGs: differentially expressed genes
DHI: Danhong injection
IRI: ischemic/reperfusion injury
KEGG: Kyoto Encyclopedia of Genes and Genomes
MCAO: middle cerebral artery occlusion
qRT-PCR: quantitative reverse transcription-polymerase chain reaction
TLR: Toll-like receptor
